# Sphingosine Kinase 1 Serves as a Pro-Viral Factor by Regulating Viral RNA Synthesis and Nuclear Export of Viral Ribonucleoprotein Complex upon Influenza Virus Infection

**DOI:** 10.1371/journal.pone.0075005

**Published:** 2013-08-30

**Authors:** Young-Jin Seo, Curtis J. Pritzl, Madhuvanthi Vijayan, Kavita Bomb, Mariah E. McClain, Stephen Alexander, Bumsuk Hahm

**Affiliations:** 1 Departments of Surgery & Molecular Microbiology and Immunology, University of Missouri-Columbia, Columbia, Missouri, United States of America; 2 Division of Biological Sciences, University of Missouri-Columbia, Columbia, Missouri, United States of America; The Scripps Research Institute, United States of America

## Abstract

Influenza continues to pose a threat to humans by causing significant morbidity and mortality. Thus, it is imperative to investigate mechanisms by which influenza virus manipulates the function of host factors and cellular signal pathways. In this study, we demonstrate that influenza virus increases the expression and activation of sphingosine kinase (SK) 1, which in turn regulates diverse cellular signaling pathways. Inhibition of SK suppressed virus-induced NF-κB activation and markedly reduced the synthesis of viral RNAs and proteins. Further, SK blockade interfered with activation of Ran-binding protein 3 (RanBP3), a cofactor of chromosome region maintenance 1 (CRM1), to inhibit CRM1-mediated nuclear export of the influenza viral ribonucleoprotein complex. In support of this observation, SK inhibition altered the phosphorylation of ERK, p90RSK, and AKT, which is the upstream signal of RanBP3/CRM1 activation. Collectively, these results indicate that SK is a key pro-viral factor regulating multiple cellular signal pathways triggered by influenza virus infection.

## Introduction

Influenza virus infection causes substantial medical cost, hospitalizations, and deaths every year worldwide. In the United States, it is estimated that between 3,000 and 49,000 people die annually due to the infection with seasonal influenza viruses [1]. Furthermore, the emergence of antiviral drug-resistant seasonal influenza viruses and highly pathogenic influenza viruses such as avian influenza A (H5N1) and 2009 pandemic influenza (H1N1) viruses continues to threaten humankind [2–5]. However, therapeutic maneuvers against influenza are often limited due to the high genetic variability of influenza virus that leads to the escape of host immune surveillance and the resistance to anti-viral drugs. Therefore, there are needs for identifying new therapeutic targets that are key factors of influenza virus replication and minimally affected by genetic mutation of influenza virus.

Influenza virus is a single-stranded RNA virus belonging to the *Orthomyxoviridae* family and its genome consists of eight distinct RNA segments which encode 11-12 proteins [6–8]. The replication process of influenza virus involves numerous interactions of the virus with host cellular signaling components for efficient virus propagation. At the early stage of infection, viral hemagglutinin rapidly activates protein kinase C [9], which is crucial for the entry of influenza virus into target cells [10,11]. Activation of the PI3K/AKT signaling pathway is also known to contribute to the entry step of influenza virus infection [12]. Viral genomic RNAs are subsequently released into the cytoplasm and rapidly imported into the nucleus. Then, viral genomic RNAs are transcribed to mRNAs that encode viral proteins. Also, genomic RNAs are amplified via the replication process. The synthesis of viral RNAs requires activation of NF-κB signaling [13]. In the late phase of influenza virus replication cycle, viral ribonucleoprotein (RNP) complexes are assembled in the nucleus and exported to the cytoplasm, which is another critical step of virus replication [14]. Nuclear export of viral RNPs and virus production are strongly prevented by treatment with an inhibitor specific to the cellular protein chromosome region maintenance 1 (CRM1) [15–17], indicating an important role of CRM1 in influenza viral propagation. In addition, the activation of ERK MAPK and PI3K/AKT signaling pathways is necessary for the transport of influenza viral RNP complexes to the cytoplasm [18–20].

Sphingosine kinase (SK) controls the level of bioactive lipid molecules by generating sphingosine 1-phosphate (S1P) from sphingosine [21,22]. The enzyme is known to regulate important cellular signaling pathways including NF-κB, ERK MAPK, and PI3K/AKT under multiple experimental conditions [23,24]. Therefore, SK1 influences diverse cellular physiologic conditions or disease progression [25–28]. For instance, SK1 increases resistance to cellular stress such as anti-cancer drug-induced apoptosis or serum deprivation [29,30].

In our previous studies, the overexpression of SK1 made cells more susceptible to influenza virus infection and its cytopathic effects, indicating its advantageous role in influenza virus propagation [31,32]. Here, we have found that Influenza virus increases expression/activation of SK1 and inhibition of SK impairs influenza virus propagation. SK inhibition suppresses the activation of NF-κB to reduce viral RNA synthesis. Further, SK inhibition interferes with the activation of ERK and AKT, leading to the inhibition of CRM1/RanBP3-mediated nuclear export of viral RNP complexes. This study unveils new mechanisms of how SK regulates cellular signaling pathways for successful influenza virus replication. 

## Materials and Methods

### Virus and cells

Influenza A/WSN/33 virus (H1N1) was provided by Yoshihiro Kawaoka (University of Wisconsin-Madison) and used in this study. Influenza A/Hong Kong/8/68 (H3N2) virus was purchased from American Type Culture Collection (ATCC). For the titration of viruses, at various times after infection, the supernatants containing released viruses were harvested. Virus titer was determined on Madin-Darby Canine Kidney (MDCK) epithelial cells by a plaque assay [33]. MDCK cells were provided by Michael B.A. Oldstone (the Scripps Research Institute) and maintained in Minimum Essential Medium Eagle (MEM, Mediatech). Human lung epithelial A549 cells were provided by Shan-Lu Liu (University of Missouri-Columbia) and maintained in Dulbecco’s Modified Eagle’s Medium (DMEM, Mediatech). Human embryonic kidney (HEK293) and SK1-overexpressing HEK293 cells were maintained in DMEM [29]. Cells were cultured in CO_2_ incubator at 37 °C and all media were supplemented with 10% fetal bovine serum (HyClone) and penicillin (100 U/ml) / streptomycin (100 μg/ml) (Invitrogen).

### Specific Inhibitors of cellular signaling pathways/metabolism

To inhibit SK activity, cells were treated with SK-specific inhibitors SKI-II (Sigma-Aldrich), N,N, -dimethylsphingosine (DMS, Cayman Chemical), or SK1-I (TOCRIS) or solvent (DMSO for SKI-II and DMS; water for SK1-I) as a control. Leptomycin B (LMB, Sigma-Aldrich) was used to inhibit CRM1. U0126 (Cell Signaling) and LY294002 (Calbiochem) were used to inhibit the activation of ERK MAPK and PI3K/AKT, respectively. Specific inhibitors of protein synthesis (cycloheximide, CHX) and the NF-κB signaling pathway (BAY11-7082, BAY) were purchased from Sigma-Aldrich.

### Western blot analysis

Specific antibodies against influenza viral NP (ab43821), M1 (ab22396), and M2 (ab5416) were purchased from Abcam; influenza viral NS1 (sc-130568), IKKαβ (sc-7607), α-tubulin (sc-23948), and actin (sc-1616) were purchased from Santa Cruz Biotechnology; histone H2B (HP4291) and pSK1 (SP1641) were purchased from ECM Biosciences; influenza viral NS2 (A01499) was purchased from GenScript; RanBP3 (PA1-084) was purchased from Affinity BioReagents; ERK (9102), pERK (4370), AKT (9272), pAKT (Ser473, 4060), SK1 (3297), pIKKαβ (2697), p65 (8242), p-p65 (3033), p90RSK (9355), p-p90RSK (9335), pRanBP3 (9380), and GAPDH (5174) were purchased from Cell Signaling Technology. Total proteins were extracted with RIPA buffer supplemented with inhibitors blocking proteases and phosphatases, and then normalized using a Bradford assay. For nuclear/cytosolic protein fractionation, NE-PER® Nuclear and Cytoplasmic Extraction Reagents (Thermo Scientific) were used according to the manufacturer’s instructions. For detection of internalized viral M1 protein, A549 cells were infected with influenza virus A/WSN/33 at 10 MOI at 4^°^C for 1 hr followed by an incubation at 37^°^C for an additional 1 hr. After washing with PBS/HCl (pH 1.3) and PBS, cells were lysed in RIPA buffer as described by Eierhoff et al [34]. Equal protein samples (10-20 μg each) were resolved on a 10-12% SDS-PAGE gel and transferred to a nitrocellulose membrane (PROTRAN-NC, Whatman). Membrane bound antibodies were detected by enhanced chemiluminescence (Thermo Scientific). All the presented data were repeated at least twice with independent experimental settings.

### RNA interference

Small interfering RNAs (siRNA) targeting SK1 (siSK1) and RanBP3 (siRanBP3) were synthesized by Qiagen and Dharmacon, respectively. Scramble siRNA was purchased from Dharmacon and used as a control. Cells were transfected with 50 (siRANBP3) or 100 (siSK1) nM siRNA using Lipofectamine RNAiMAX (Invitrogen) according to the manufacturer’s instructions. Cells were then used for further experiments at day 3 after transfection. Knockdown of SK1 and RanBP3 expression was verified by Western blot analysis. The experiment was independently repeated three times with similar results.

### NF-κB luciferase reporter assay

MDCK cells were co-transfected with 100 ng of NF-κB promoter-luciferase reporter plasmid (pGL3-NF-κB) [35] and 20 ng of Renilla luciferase plasmid (pRL-CMV) which was used as a transfection control. After 12 hrs, cells were treated with solvent or SKI-II (10 µM) and infected with influenza A/Hong Kong/8/68 virus at 5 MOI for 7 hrs or were transfected with influenza viral RNA (100 ng) for 7 hrs. Influenza virus RNA was purified from influenza A/WSN/33 virus stock using Tri-reagent (Sigma-Aldrich) according to the manufacturer’s description and treated with DNase I (Fermentas) to remove any contaminating DNAs. After infection with influenza virus or transfection with influenza viral RNA, cell lysates were analyzed for Firefly and Renilla luciferase activity according to manufacturer’s instructions of the Dual-Luciferase Reporter Assay System (Promega).

### Immunocytochemistry

Cells were plated on 4 or 8-well chamber slides (Nunc). The following day, cells were treated with the SK inhibitor and infected with influenza virus. At indicated time points, cells were fixed in 4% paraformaldehyde (Fisher) and then permeabilized in 0.5% Triton X-100 (Sigma) for 10 min at room temperature. Cells were blocked in 1% BSA for 2 hours and then incubated with p65 (1: 200, Cell Signaling) antibody or antibodies specific for viral proteins: NP, M1, M2 (1:200, Abcam), NS1, or NS2 (1:200, Santa Cruz) overnight at 4^°^C. Cells were incubated with Alexa Fluor® 488-conjugated anti-mouse IgG (1:1000, Invitrogen) or Alexa Fluor 546®-conjugated anti-rabbit IgG (1:1000, Invitrogen) for 2 hrs, followed by staining with DRAQ5 dye (300 nM, Thermo Scientific) for 15 min at room temperature. The images were obtained on a Zeiss LSM 510 META confocal microscopy and analyzed with Zeiss LSM Image Browser software. Representative fields are shown: each image was selected from 5–10 different fields. Results were equivalent in repeated experiments.

### Real Time PCR

Total cellular RNA was purified using Tri-reagent (Sigma-Aldrich) according to the manufacturer’s description and treated with DNase I (Fermentas) to remove contaminating DNAs. Total RNA was reverse-transcribed with oligo dT (Promega) for GAPDH or primers for (-)-sense (5’-TGC TTC CAA TGA AAA CAT GG-3’) or (+)-sense (5’-GCC CTC TGT TGA TTG GTG TT-3’) RNA of influenza viral NP or (-)-sense (5’-GTT GGG AGA AGA GCA ACA GC-3’) or (+)-sense (5’-AAT TAT TGC TTC GGC AAT CG-3’) RNA of influenza viral PB2 and then analyzed by qPCR using gene-specific primer sets. Primers for viral NP (5’-TGC TTC CAA TGA AAA CAT GG-3’, 5’-GCC CTC TGT TGA TTG GTG TT-3’), viral PB2 (5’-GTT GGG AGA AGA GCA ACA GC-3’, 5’-AAT TAT TGC TTC GGC AAT CG-3’), and GAPDH (5’-TCA CCA CCA TGG AGA AGG-3’, 5’-GAT AAG CAG TTG GTG GTG CA-3’) were used. Quantitative real-time PCR reactions were performed with SYBR Green I chemistry using an ABI 7900 HT real time PCR instrument. The authenticity of the PCR products was verified by melting curve analysis. cDNA quantities were normalized to GAPDH RNA quantities measured in the same samples. The experiment was independently repeated twice with similar results.

### Statistical Analysis

All error bars represent mean ± standard error of the mean (SEM), and averages were compared using a bidirectional, unpaired Student’s *t*-test. 

## Results

### SK1 activation is enhanced by influenza virus infection and critical for the virus replication

**Figure 1:**
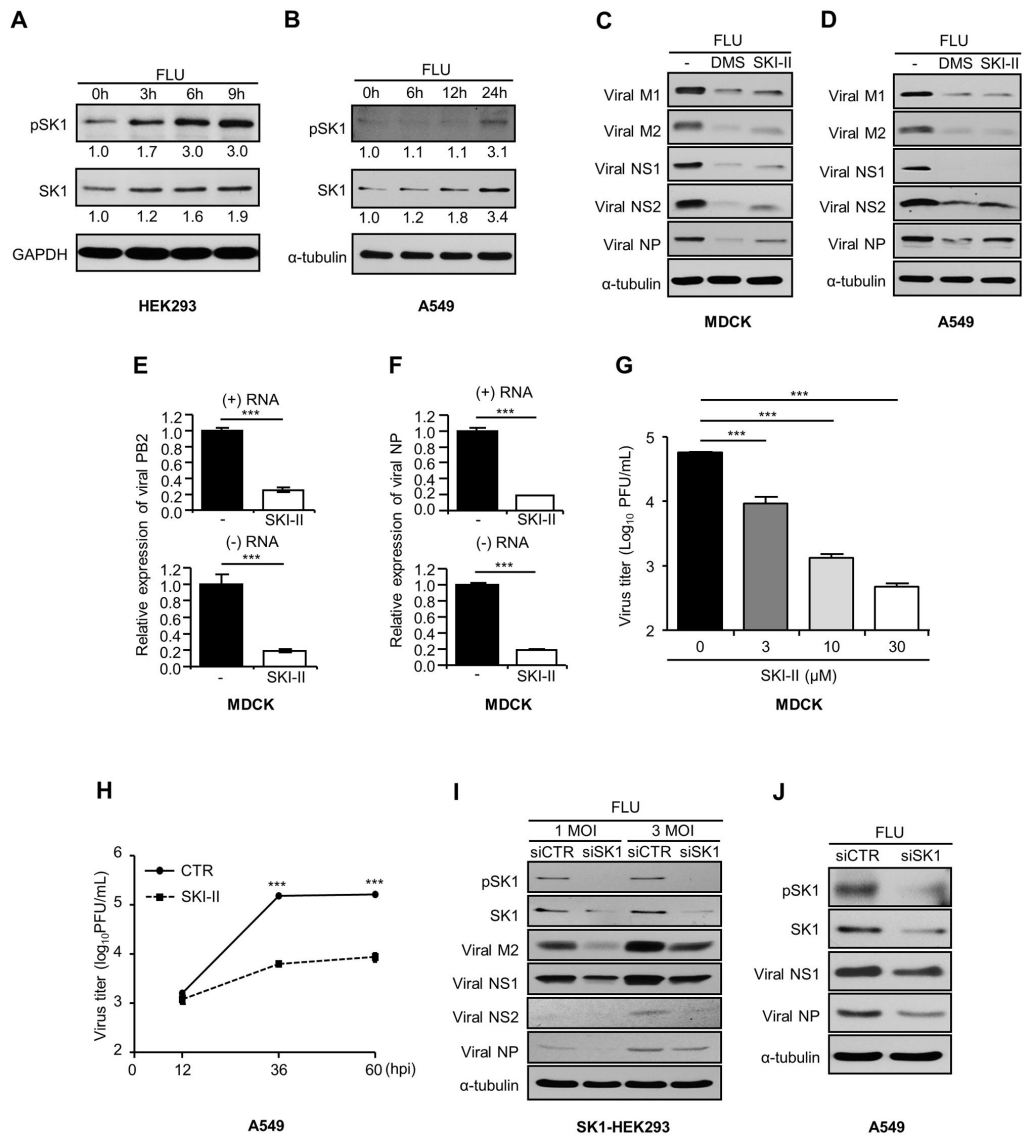
Influenza virus increases SK1 activation, which is critical for viral replication. (A and B) HEK293 (A) or A549 (B) cells were infected with influenza A/WSN/33 virus (FLU) at an MOI of 3 for the indicated times. The levels of pSK1 or SK1 were analyzed by Western blotting. GAPDH or α-tubulin was used as internal loading control. The relative intensities for each band of pSK1 and SK1 were determined based on the control protein expression by densitometery and depicted below each blot. The relative level of protein at 0 hr was set as 1.0. (C and D) MDCK (C) or A549 (D) cells were treated with solvent (DMSO; -), DMS (5 µM), or SKI-II (10 µM) upon influenza virus infection at an MOI of 1. Cell lysates were used for Western blot analysis to detect viral proteins M1, M2, NS1, NS2, NP, and α-tubulin at 12 hpi. (E and F) MDCK cells were treated with solvent (-) or SKI-II upon influenza virus infection at an MOI of 5. The expression of (+) or (-) RNA of viral RNA polymerase subunit PB2 (E) or viral NP (F) was analyzed by real-time quantitative PCR (qPCR) at 5 hpi. Three reactions per sample were carried out. Values are means ± SEM of three reactions per sample. ***p<0.001. (G) MDCK cells were treated with solvent or SKI-II (3, 10, or 30 µM) and infected with influenza virus at an MOI of 0.01. At 24 hpi, a plaque assay was performed to determine virus titer (plaque forming unit, PFU/mL) at each condition. Values are means ± SEM. N = 3/group. ***p<0.001. (H) A549 cells were treated with solvent or SKI-II (10 µM) and infected with influenza virus at an MOI of 0.1. At 12, 36, or 60 hpi, viral titers (PFU/mL) were determined by a plaque assay. Values are means ± SEM (n=3). ***p<0.001. (I and J) SK1-overexpressing HEK293 (I) or A549 (J) cells were transfected with scramble siRNA control (siCTR) or siRNA targeting SK1 (siSK1). After 3 days, the cells were infected with influenza virus at an MOI of 1 (I) or 3 (I and J). At 12 hpi, Western blot analysis was performed to detect pSK1, SK1, viral proteins M2, NS1, NS2, NP, and α-tubulin.

SK1’s stimulatory effect on virus propagation [31,32] prompted us to investigate if influenza virus infection regulates SK1 in cells. Following influenza A/WSN/33 virus (H1N1) infection, the level of SK1 protein increased in human embryonic kidney (HEK) 293 cells (Figure 1A) and human lung epithelial A549 cells (Figure 1B). Since phosphorylation of SK1 at serine residue (Ser255) confers its activation [36], the phosphorylation of SK1 was monitored following virus infection. Elevation of SK1 phosphorylation was detected after influenza virus infection of HEK293 (Figure 1A) and A549 cells (Figure 1B). Thus, we conclude that influenza virus infection increases both SK1 level and activation, which could be beneficial for the virus by promoting its own replication.

Since the amplification of influenza virus was promoted when cells were engineered to overexpress SK1 [31], we determined if inhibition of SK1 in unmodified cells suppresses viral replication. To this end, cells were left untreated or co-treated with specific SK inhibitors, dimethylsphingosine (DMS) [27,37,38] or sphingosine kinase inhibitor II (SKI-II) [27,39], upon influenza virus infection. SK inhibitors markedly suppressed expression of viral proteins including matrix proteins (M1, M2), non-structural proteins (NS1, NS2), and nucleoprotein (NP) in Madin-Darby Canine Kidney (MDCK) (Figure 1C), A549 (Figure 1D), HEK293, or human bronchoalveolar NCI-H358 cells (data not shown) in Western blot analyses. The decreased expression of virus proteins is not due to the altered cell viability, since the SK inhibitors did not display cytotoxicity (Figure S1). These results represent a critical role of SK during influenza virus replication.

Next, we determined whether the diminished viral protein expression by SK inhibition is associated with impairment of viral RNA synthesis. The synthesis of negative sense (-) RNA (viral genomic RNA) and positive sense (+) RNAs was analyzed using strand-specific reverse transcription followed by real-time quantitative PCR (qPCR) with PB2 subunit of viral RNA polymerase (PB2) or viral NP-specific oligonucleotides. When the RNA amplification was assessed at 5 hours post-infection (hpi), SKI-II treatment strongly suppressed the synthesis of both (+) RNA and (-) RNA (Figure 1E and 1F). The results suggest that decreased viral protein expression could be caused by a reduced viral mRNA pool available for translation processes. Reduced amounts of viral proteins directing replication/transcription could also be responsible for the inhibition of viral RNA synthesis. Therefore, SK inhibition interferes with the generation of both viral proteins and RNA genome required for the formation of new viral particles. Indeed, treatment with the SK inhibitor SKI-II significantly decreased production of infectious influenza viruses from MDCK cells in a dose dependent manner (Figure 1G). Strong inhibition of virus production by SKI-II was also observed at 36 and 60 hpi in A549 cells (Figure 1H). Similarly, the SK inhibitor DMS effectively diminished progeny virus production from MDCK, NCI-H358, A549, or HEK293 cells (data not shown). We further utilized a small interfering RNA (siRNA) approach to confirm our observations. Specific siRNA targeting of SK1 down-regulated the expression of SK1 and pSK1 upon influenza virus infection. Consequently, the expression of viral proteins was noticeably decreased in both SK1-overexpressing HEK293 cells (Figure 1I) and A549 cells (Figure 1J) compared to control cells. When cells were infected with influenza, A/Hong Kong/8/68 virus (H3N2), increased SK1 and pSK1 levels were observed (Figure S2A) and SK inhibitors such as SKI-II and SK1-I [23] suppressed the expression of viral proteins (Figures S2B and S2C). Collectively, these data demonstrate that inhibition of SK expression or activation suppresses influenza virus replication.

### SK inhibition does not impair the entry step of influenza virus infection

**Figure 2:**
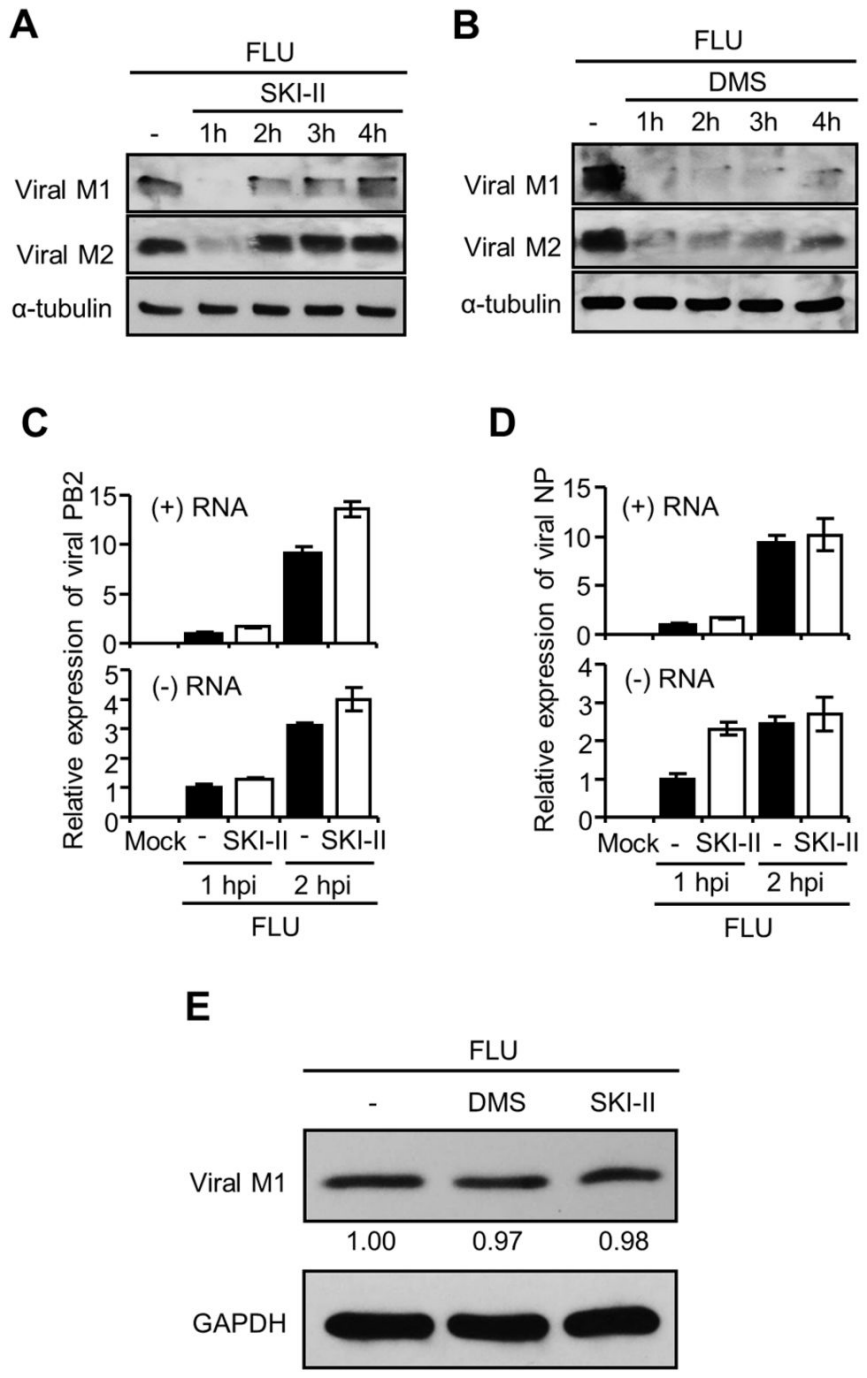
SK inhibition does not interfere with the viral entry step. (A and B) MDCK cells were infected with influenza virus (1 MOI) for 1 hr, followed by washing with PBS. Cells were then treated with SKI-II (10 µM) (A) or DMS (5 µM) (B) at 1, 2, 3, or 4 hpi. The expression of viral proteins M1 and M2 was detected by Western blot analysis at 8 hpi. (C and D) MDCK cells were treated with solvent or SKI-II (10 µM) upon influenza virus infection at an MOI of 1. The synthesis of (+) or (-) viral PB2 (C) or NP (D) RNA was analyzed by qPCR at 1 or 2 hpi. The RNA level at 1 hpi was set as 1.0. Values are means ± SEM of three reactions per sample. (E) A549 cells were treated with solvent, DMS (5 µM), or SKI-II (10 µM) and infected with influenza virus at an MOI of 10. The virus was allowed to attach to the cells at 4^°^C for 1 hr and the cells were incubated at 37^°^C for an additional 1 hr. Western blot analysis was performed to detect internalized viral M1 and GAPDH. The relative intensities for each band of viral M1 are shown.

It is conceivable that SK inhibition impairs viral entry steps including viral attachment and endocytosis leading to the inhibition of virus propagation. To examine this possibility, we performed three experiments. Firstly, cells were infected with influenza virus for 1 hour and washed to remove unattached virus particles. After 1 to 4 hpi, SK inhibitor was supplied to the infected cells. Even after the virus had adsorbed and entered the cells, SKI-II and DMS displayed an inhibitory activity on viral protein expression (Figure 2A and 2B). This means that SK inhibition could impair viral replication at the post-entry level. However, the result does not fully exclude the idea that SK inhibition affects the viral entry step. Therefore, in a second set of experiments, cells were left untreated or co-treated with SKI-II upon influenza virus infection and after 1 or 2 hpi, viral RNAs were measured to assess the initial status of RNAs before massive RNA amplification occurs. SKI-II did not inhibit the delivery or initial synthesis of (+) RNAs and (-) RNAs of viral PB2 (Figure 2C) and NP (Figure 2D) at early times of infection (1-2 hpi). We found that viral RNAs were markedly increased between 3 and 5 hpi in MDCK cells (20-50 fold, data not shown) and, as shown in Figure 1E and 1F, SK inhibition strongly suppressed RNA synthesis at 5 hpi. Therefore, SK inhibition inhibited the synthesis of positive and negative strands of RNAs between 2 and 5 hpi, but did not impair the delivery or production of viral RNAs between 0 and 2 hpi. Lastly, the effect of SK inhibition on influenza virus entry was assessed by detection of internalized viral M1 in Western blot analysis. Eierhoff et al [34] reported that viral M1 detected at one hpi was not newly synthesized protein, as demonstrated by the treatment with a protein synthesis inhibitor. The treatment with the SK inhibitor DMS or SKI-II did not decrease viral M1 level compared to the solvent treated cells at one hpi (Figure 2E). Therefore, these data suggest that SK inhibition does not interfere with the early stage of influenza virus infection including the viral entry into the cells before viral RNA synthesis begins in earnest.

### Influenza virus infection-induced NF-κB activation is repressed by SK inhibition

**Figure 3:**
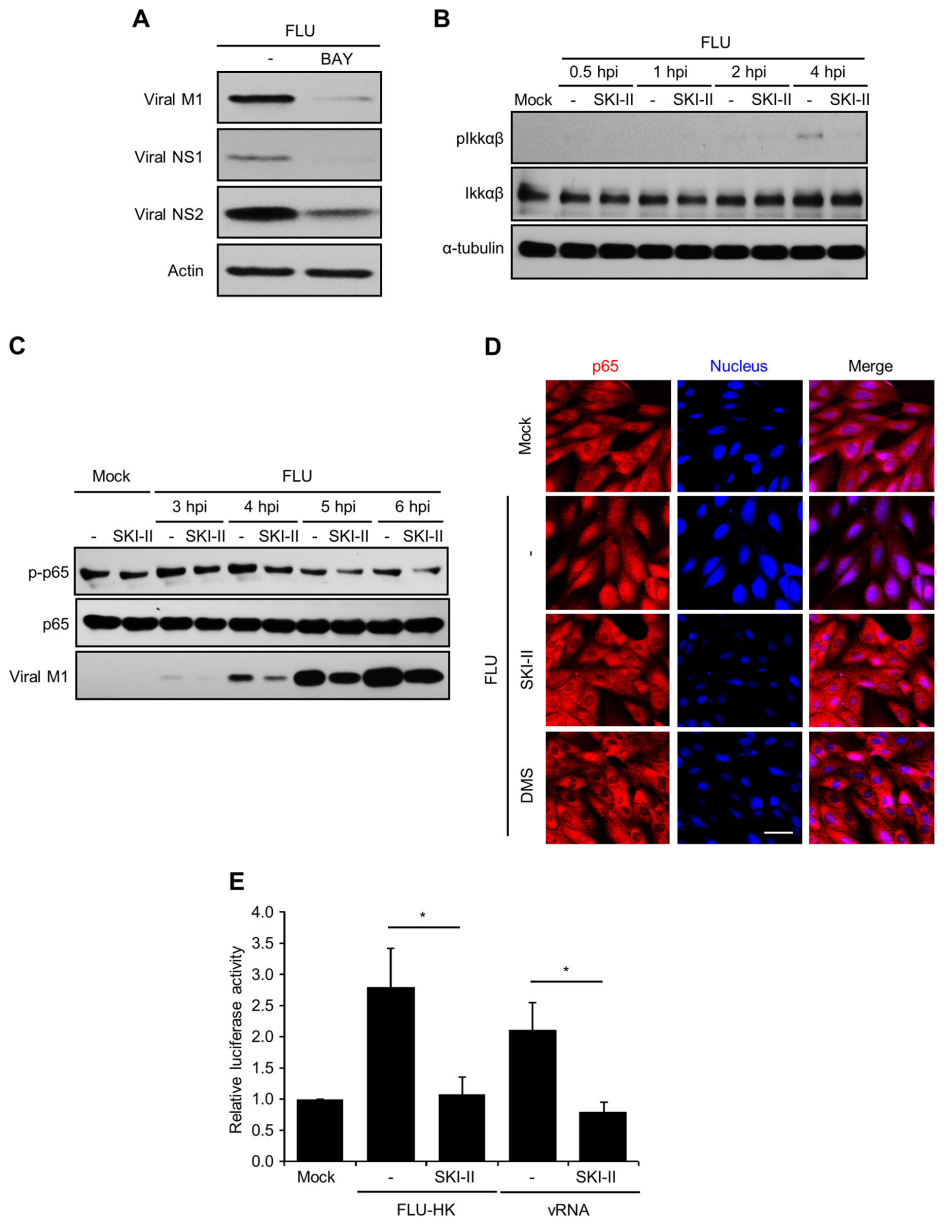
SK inhibition suppresses influenza virus replication by impairing activation of the NF-κB signaling pathway. (A) MDCK cells were left untreated or treated with BAY11-7082 (2.5 µM) and infected with influenza virus at an MOI of 1. The expression of viral proteins M1, NS1, NS2, and actin was assessed by Western blotting at 7 hpi. (B) MDCK cells were treated with solvent or SKI-II (10 µM) and uninfected (Mock) or infected with influenza virus at an MOI of 3. At 0.5, 1, 2, or 4 hpi, Western blot analysis was performed to detect pIKKαβ, IKKαβ, and α-tubulin. (C) MDCK cells were treated with solvent alone or SKI-II (10 µM) and uninfected (Mock) or infected with influenza virus (3 MOI). At 3, 4, 5, or 6 hpi, Western blotting was performed to detect p-p65, p65, and viral M1. (D) MDCK cells were mock-infected or infected with influenza virus at an MOI of 3. They were fixed, permeabilized, and stained with antibodies against NF-κB subunit p65 (red) at 4 hpi and DRAQ5 dye to detect nuclei (blue). Representative confocal images are shown. Scale bar = 50 µm. (E) MDCK cells were co-transfected with NF-κB luciferase reporter plasmid and control Renilla luciferase plasmid. After 12 hrs, cells were treated with solvent alone or SKI-II (10 µM) upon influenza A/Hong Kong/8/68 virus infection at 5 MOI for 7 hrs or were transfected with influenza viral RNA (100 ng) for 7 hrs. Cell lysates were analyzed for luciferase activity with a luminometer. Relative luciferase activities are shown. Values are means ± SEM (n=3). *p<0.05.

These findings led us to speculate that SK regulates cellular machinery that is crucial for viral RNA replication/transcription. Previously, influenza virus was shown to activate NF-κB signaling which then increases viral RNA synthesis [13]. As reported, an NF-κB-specific inhibitor (BAY11-7082) strongly suppressed viral protein expression in MDCK cell at 7 hpi (Figure 3A). Influenza virus infection increased phosphorylation of IKKαβ, an upstream signaling molecule of NF-κB, at 4 hpi in MDCK cells. The treatment with SKI-II strikingly reduced virus-induced phosphorylation of IKKαβ (Figure 3B). Similarly, phosphorylation of p65, a subunit of NF-κB was impaired by SKI-II upon influenza virus infection (Figure 3C). In a confocal microscopic analysis, influenza virus infection induced nuclear localization of the NF-κB p65 subunit, which is indicative of NF-κB activation. SK inhibitors DMS or SKI-II noticeably reduced the nuclear localization of p65 (Figure 3D), consistent with the altered phosphorylation of NF-κB signal components. The translocated NF-κB is known to bind its DNA promoter to induce gene transcription. Therefore, activation of the NF-κB promoter and its inhibition by SKI-II was measured by a luciferase reporter assay. SKI-II strongly inhibited influenza virus-induced or viral RNA-mediated transcriptional activity of NF-κB (Figure 3E). Thus, SK inhibitor treatment reduces virus-induced NF-κB activation, which could lead to the inhibition of viral RNA synthesis.

### SK inhibition suppresses nuclear export of influenza viral RNP complex

**Figure 4:**
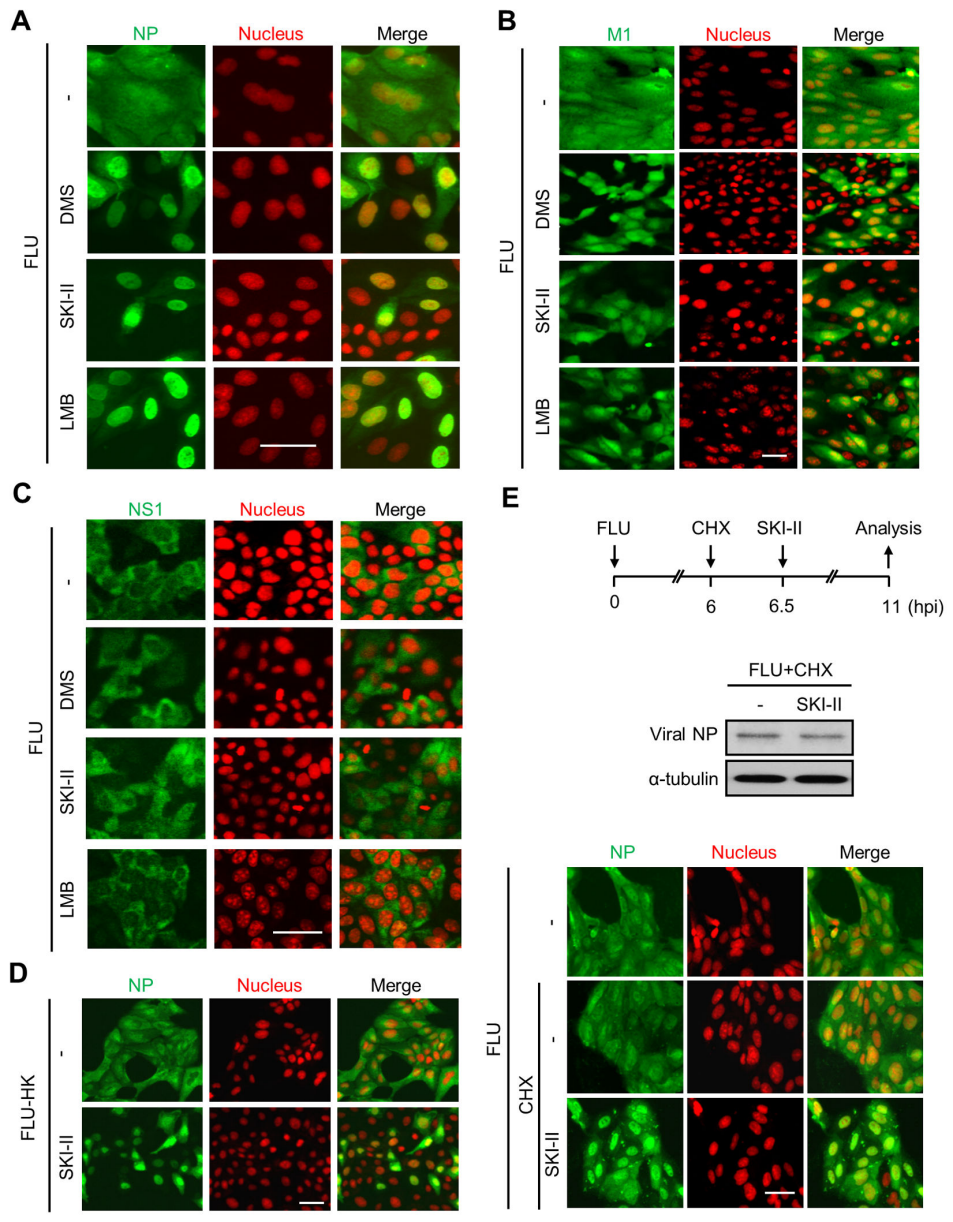
SK inhibitor treatment interferes with nuclear export of influenza viral RNP complex. (A) MDCK cells were treated with solvent (-), DMS (5 µM), or SKI-II (10 µM), or leptomycin B (LMB, 10 ng/mL) and infected with influenza virus at 1 MOI for 12 hrs. Cells were then fixed, permeabilized, and stained with antibodies against viral NP (green) and with DRAQ5 dye to detect nuclei (red). (B and C) MDCK cells were treated with solvent, DMS (5 µM), SKI-II (10 µM), or LMB (10 ng/mL) and infected with influenza virus at an MOI of 1 for 12 hrs. After staining with antibodies against viral M1 (B) or NS1 (C), viral proteins (green) were visualized by confocal microscopic analysis. DRAQ5 dye was used to stain nuclei (red). (D) MDCK cells were treated with solvent or SKI-II (10 µM) and infected with influenza A/Hong Kong/8/68 (FLU-HK) virus at 1 MOI for 10 hrs. (E) MDCK cells were infected with influenza virus at an MOI of 1 and then treated with cycloheximide (CHX) at 6 hpi or left untreated. The cells were then treated with solvent or SKI-II at 6.5 hpi for an additional 4.5 hrs (top panel). For viral NP and α-tubulin detection, Western blot analysis was performed (middle panel). Viral NP expression was analyzed by confocal microscopy (bottom panel). Cells were stained with DRAQ5 dye to detect nuclei. Scale bar = 50 µm.

During influenza virus infection, the nuclear export of viral RNP complexes is one of the key steps for its replication [14]. We have determined whether SK inhibition has any impact on nuclear export of viral RNP complex. The export of viral RNP complexes was reported to be dependent on the CRM1-mediated nuclear export pathway [16]. In agreement with this, CRM1 inhibitor leptomycin B (LMB) treatment completely blocked nuclear export of viral NP, which is a major component of the viral RNP complex, when analyzed by confocal microscopy (Figure 4A). Intriguingly, SK inhibitors such as DMS, SKI-II (Figure 4A), and SK1-I (Figure S3A) also repressed nuclear export of viral NP when compared to virus-infected control cells. The inhibitor-induced nuclear retention of viral NP was confirmed by separately fractionating cytosolic and nuclear proteins from MDCK cells. In Western blot analyses, consistent with the result in Figure 4A, the relative level of cytosolic viral NP compared to the nuclear NP level in SKI-II-treated MDCK cells was approximately 2 fold lower than that in untreated control cells (Figure S3B). Furthermore, nuclear export of viral M1 (Figure 4B) and NS2 (Figure S3C) was partially inhibited by SK inhibitor treatment when analyzed by confocal microscopic or Western blot analyses (data not shown). In contrast, neither SK inhibition nor LMB treatment affected the localization of influenza viral NS1 (Figure 4C) and M2 (Figure S3D). In addition to viral NP, viral M1 [40,41] and NS2 [42] are known to play important roles in the nuclear export of influenza viral RNP complexes presumably by associating with the RNP complex, whereas NS1 and M2 do not. Further, inhibition of nuclear export of viral NP was observed when cells were infected with influenza A/Hong Kong/8/68 virus and treated with SKI-II (Figure 4D). Thus, these results indicate that inhibition of SK suppresses the nuclear export of influenza viral RNP complexes.

Suppressed nuclear export of viral RNP complex could simply be due to the reduced amount of viral proteins caused by SK inhibitor treatment. In other words, viral proteins may not exist in sufficient amounts in SK-inhibited cells to actively stimulate the host cellular signaling pathways that are critical for nuclear export of viral RNP complexes. To further investigate this possibility, MDCK cells were treated with a protein synthesis inhibitor cycloheximide (CHX) to block additional viral protein synthesis at 6 hpi. After 30 min, cells were left untreated or treated with SKI-II for an additional 4.5 hours (top panel of Figure 4E). Although SKI-II treatment following CHX treatment did not change viral NP expression in Western blotting (middle panel of Figure 4E), nuclear export of viral NP was strongly impaired by SK inhibition (bottom panel of Figure 4E) when viral NP localization was analyzed by a confocal microscopy. Thus, we could conclude that SK inhibition suppresses nuclear export of viral RNP complexes, which is independent of SK inhibition-mediated suppression of viral protein expression.

### Inhibition of SK reduces activation of ERK MAPK and PI3K/AKT signaling pathways to inhibit RanBP3 phosphorylation upon influenza virus infection

**Figure 5:**
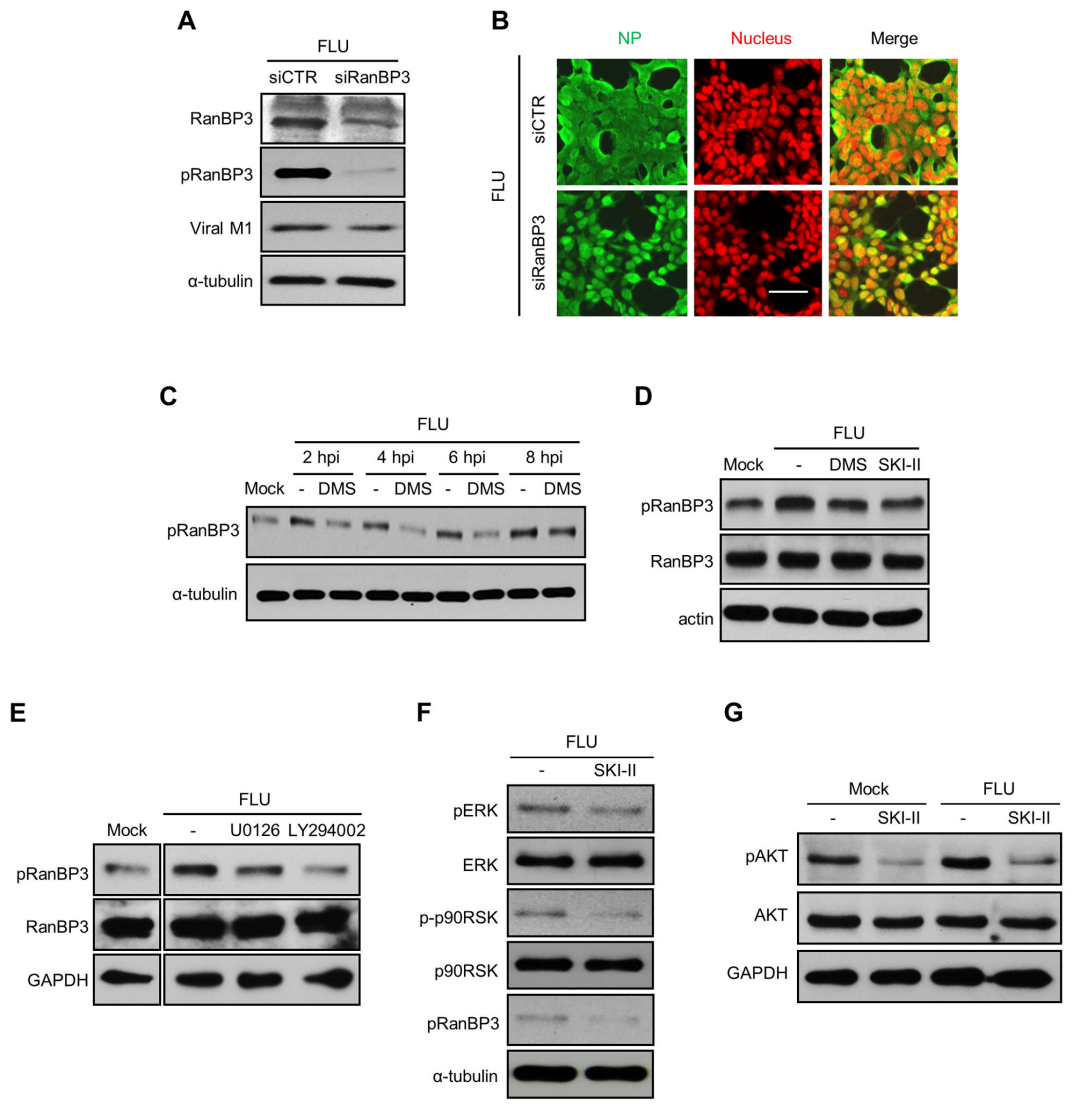
SK inhibition impairs virus-induced activation of ERK/p90RSK/AKT to inhibit RanBP3-mediated nuclear export of viral NP. (A and B) HEK293 cells were transfected with scramble siRNA (siCTR) or siRNA targeting RanBP3 (siRanBP3); then the cells were infected with influenza virus at an MOI of 0.01 (A) or 1 (B). RanBP3, pRanBP3, viral M1, and α-tubulin were detected by Western blotting at 30 hpi (A). Viral NP (green) and nuclei (DRAQ5 dye, red) were visualized by confocal microscopy at 12 hpi (B). (C and D) MDCK cells were treated with solvent, DMS (5 µM), or SKI-II (10 µM) and infected with influenza virus at an MOI of 5 for the indicated times (C) or 6 hrs (D). Cell lysates were used for Western blot analysis to detect pRanBP3, RanBP3, α-tubulin, or actin. (E) MDCK cells were left untreated or treated with U0126 (10 µM) or LY294002 (10 µM) and infected with influenza virus at 3 MOI for 6 hrs. Western blot analysis was performed to detect pRanBP3, RanBP3, and GAPDH. (F) MDCK cells were treated with solvent alone or SKI-II (10 µM) and infected with influenza virus at an MOI of 1. Western blot analysis was performed to detect pERK, ERK, p-p90RSK, p90RSK, pRANBP3, or α-tubulin at 12 hpi. (G) MDCK cells were treated with solvent or SKI-II (10 µM) and mock-infected or infected with influenza virus at an MOI of 3. At 8 hpi, pAKT, AKT, and α-tubulin were detected by Western blot analysis.

RanBP3 often works as a cofactor of CRM1 to mediate nuclear protein export [43]. Very recently, RanBP3 activation was shown to regulate nuclear export of influenza viral RNP complexes [44]. We have also determined intrinsic roles of RanBP3 in influenza virus replication by conducting RNA interference experiments. While down-regulation of RanBP3 only minimally influenced viral protein expression (Figure 5A), it strongly impaired nuclear export of viral RNP complex (Figure 5B). The data indicate that RanBP3 is critical for the replication of influenza virus, which is consistent with the recently published results [44]. Next, we have determined whether SK inhibition has any effect on RanBP3 phosphorylation, which is indicative of its activation [45]. Influenza virus infection increased the activation of RanBP3 and the SK inhibitor DMS or SKI-II reduced phosphorylation of virus-induced pRanBP3 (Figure 5C and 5D). The result indicates that SK1 is required for influenza virus-mediated phosphorylation of RanBP3. Therefore, inhibition of SK suppresses activation of RanBP3 that is crucial for promoting nuclear export of influenza viral RNP complexes.

RanBP3 is known to be phosphorylated by p90RSK, a downstream molecule of ERK MAPK and PI3K/AKT [45]. Moreover, the ERK MAPK [18] and PI3K/AKT [20] signaling pathways were shown to be required for influenza virus replication by promoting the nuclear export of influenza viral RNP complexes. The treatment with ERK MAPK (U0126) or PI3K/AKT (LY294002) pathway-specific inhibitor reduced influenza virus infection-mediated phosphorylation of RanBP3 (Figure 5E). This result indicates that ERK MAPK and PI3K/AKT are upstream of RanBP3 upon influenza virus infection. Therefore, we have examined whether SK inhibition alters influenza virus-induced activation of ERK MAPK and PI3K/AKT signaling pathways. SK inhibition reduced influenza virus-induced phosphorylation of ERK, p90RSK, and RanBP3 (Figure 5F) in MDCK cells. This supports the result of SK inhibitor-mediated suppression of ERK MAPK signaling pathway observed in leukemia cells in other experimental conditions [23]. Furthermore, influenza virus-induced phosphorylation of AKT was also decreased by SK inhibitor treatment (Figure 5G). However, SK inhibitor did not decrease the activation of p38 MAPK upon influenza virus infection (data not shown). These findings indicate that inhibition of SK reduces influenza-mediated activation of ERK MAPK and PI3K/AKT signaling pathways, leading to the inhibition of nuclear export of viral RNP complexes. 

## Discussion

Our findings highlight an important role of SK in regulating cellular signaling pathways to enhance influenza virus replication. Influenza virus infection increases expression and phosphorylation of SK1. The activated SK acts as a key pro-viral factor, triggering cellular signal cascades to aid in influenza virus replication and propagation. Two independent molecular signaling mechanisms were identified (Figure S4). Firstly, SK contributes to the activation of the NF-κB signaling pathway following influenza virus infection. This promotes rapid synthesis of viral RNAs and proteins required for the amplification of progeny viruses. Secondly, SK increases activation of ERK MAPK and PI3K/AKT signaling pathways, leading to the phosphorylation of RanBP3. The CRM1/RanBP3 export signal is activated and mediates the nuclear export of viral RNP complexes, facilitating production of infectious virus particles.

Lipids are one of the major components of the cell membrane and therefore the viral entry step could be affected by the alteration in lipid composition of the cellular membrane. For example, cholesterol influences the efficiency of entry of several viruses such as dengue virus (DENV) [46], hepatitis C virus (HCV) [47], and West Nile virus [48]. Ceramide, one of the sphingolipids, was reported to inhibit entry of human immunodeficiency virus [49] or HCV into the cells [50]. Since S1P-metabolizing enzymes regulate the level of cellular S1P and the balance of sphingolipids [51], inhibition of SK possibly affect sphingolipid formation in cell membranes and the viral entry process. However, our results indicate that the treatment with SK-specific inhibitor does not interfere with influenza viral entry into the cells (Figure 2). Rather, inhibition of SK dysregulates post-entry stages of the viral replication process such as viral RNA synthesis and the nuclear export of RNP complexes.

Following virus infection, host cells commonly activate anti-viral machineries such as the production of type I interferons (IFNs) to inhibit viral replication and spread [52]. Influenza virus-infected cells sense viral components such as viral genomic RNAs and proteins, which triggers activation of diverse cellular signaling pathways to facilitate anti-viral responses. Paradoxically, activation of several host-defensive signaling pathways was also reported to be essential for efficient replication of influenza virus. For instance, NF-κB is generally considered as a transcription factor regulating anti-viral cytokines such as type I IFNs [53,54]. However, the activation of NF-κB was also shown to be required for effective replication of influenza virus [13,55,56]. This demonstrates a bivalent role of NF-κB in influenza viral replication and host defense system. It is unclear if the temporal/spatial activation of NF-κB is important for its function during the course of infection or if NF-κB is regulated by the infection in a cell type-specific manner. Similarly, activation of PI3K/AKT signaling pathway is required for phosphorylation of IFN regulatory factor 3 to induce IFN-β expression, whereas its activation is important for both the efficient influenza viral entry process [12] and the nuclear export of viral RNP complexes [20]. Our data suggest that influenza virus activates SK1 to regulate both NF-κB and AKT signaling pathways for its survival. It is conceivable that influenza virus-induced activation of SK is a means to divert or utilize the cellular defense strategy for viral propagation. Although the NF-κB signaling was linked to the regulation of viral RNA synthesis, the pathway could control various cellular phenomena such as cellular apoptosis, cytokine regulation, and the activation of caspases [57], which could affect influenza virus replication. Therefore, it requires further investigation to thoroughly understand the mechanism for the direct and indirect effects that SK-mediated NF-κB modulation has on influenza virus replication cycle.

RanBP3 is a Ran-interacting protein with an important role in protein transport from the nucleus to the cytoplasm. While RanBP3 is known as a cofactor of CRM1 for mediating nuclear export of many proteins, RanBP3 could also conduct a CRM1-independent export of several nuclear proteins such as β-catenin, Smad2, and Smad3 [58,59]. Since the CRM1-mediated nuclear export of viral RNPs is a critical step for influenza virus replication [15–17], the possible role of RanBP3 in influenza virus replication was investigated. We found that RanBP3 regulates the transport of viral RNPs to the cytoplasm and is necessary for the replication of influenza virus, which is consistent with the report by Predicala et al [44]. This indicates that RanBP3 works in conjunction with CRM1 to regulate the nuclear export of viral RNP complexes. Furthermore, SK inhibition suppressed the phosphorylation of RanBP3 and interfered with nuclear export of viral NP, M1, and NS2, but did not affect the intracellular localization of NS1 and M2. Similar results were observed in CRM1 inhibitor-treated cells (Figures 4 and S3). Therefore, the blockade of RanBP3 activation by SK inhibition leads to dysregulation of CRM1-mediated export signal in the influenza virus-infected cells. Previously, the Raf/MEK/ERK signaling cascade was also shown to be essential for efficient export of nuclear RNP complex [18]. Further, RanBP3 phosphorylation is mediated by ERK MAPK and PI3K/AKT signaling pathways [45]. Since SK inhibition reduces the activation of ERK MAPK and AKT, virus-induced SK seems to regulate activation of ERK MAPK and PI3K/AKT signaling pathways, leading to the phosphorylation of RanBP3 to enhance nuclear export of viral RNP complexes.

Recently, several studies have addressed roles of SK1 in virus infections. NS3 of bovine viral diarrhea virus (BVDV) directly binds SK1 to inhibit its activity, leading to enhanced virus replication [60]. Similar to BVDV, SK1 activation is reduced by DENV infection, which is associated with increased apoptosis after TNF-α stimulation [61]. However, it is unclear whether SK inhibition or stimulation directly affects the DENV replication cycle. In contrast to BVDV and DENV, respiratory syncytial virus [62], human cytomegalovirus [63], and influenza virus (Figure 1A and 1B) increase the activation of SK1. Therefore, SK1 activity is differentially regulated depending on the type of viruses, presumably because viruses have developed specialized strategies to employ host cellular machinery for their own advantage. Here, we show that SK1 regulates diverse cellular signaling pathways including ERK MAPK, PI3K/AKT, and NF-κB upon influenza virus infection. Thus, additional studies for detailed mechanisms by which SK1 regulates cellular signal transduction pathways upon infections with various viruses would increase our understanding of host–virus interactions.

Although SK’s major role is the formation of S1P, exogenously supplied S1P did not significantly affect influenza viral replication in our previous study [31]. This means that the S1P receptor signaling pathway, which is triggered by the interaction of secreted extracellular S1P with the cognate S1P receptors on the cell surface, does not regulate influenza virus replication. Accumulating data indicate that intracellular S1P could display distinctive activities that are different from the functions mediated by the secreted S1P/S1P receptor signaling pathway [64]. Further, SK1 as well as intracellular S1P was reported to interact with TNF receptor-associated factor 2 (TRAF2) and increase TNF-induced NF-κB activation [24,65]. Since our results indicate that SK inhibition impairs NF-κB signaling upon influenza virus infection (Figure 3), SK inhibition possibly affects the TNF-mediated NF-κB signaling pathway following influenza virus infection. This requires further investigation.

Recently, exogenously administered S1P receptor agonist was reported to attenuate cytokine/chemokine responses upon pathogenic influenza virus infection [66–68]. Our data indicate that influenza virus enhances SK1 expression/activation, which could increase the level of intracellular S1P. SK1 activation was shown to be important for LPS-induced IL-6 production and TNF-induced NF-κB activation [24,65,69]. Further, S1P/SK1 was implicated in exacerbation of inflammatory responses occurring in the ulcerative colitis and inflammatory bowel disease models [27,70]. Therefore, it needs to be further studied if influenza virus-induced SK activation affects the pulmonary inflammation and resultant lung injury upon infection.

Collectively, this study helps us understand mechanisms by which influenza virus modulates SK activation which in turn controls intricate cellular signals for virus propagation. Further, the identification of new cellular factors activated by influenza virus infection such as SK and the linkage of these signaling pathways could provide a basis for developing novel therapeutic interventions to treat viral diseases. 

## Supporting Information

Figure S1 **SK inhibitor does not decrease cell viability.** MDCK (A) or A549 (B) cells were treated with solvent (DMSO), DMS (5µM), or SKI-II (10 µM) for 18 (A) or 24 (B) hrs. Cellular viability was monitored by using a trypan blue exclusion assay. The total number of cells in each treatment group was set as 100% and the percentages of live cells are shown. Values are means ± SEM (n=3). NS = not significant.
(TIF)

Figure S2 **SK inhibitor suppresses the replication of influenza A/Hong Kong/8/68 virus.** (A) HEK293 cells were uninfected (Mock) or infected with influenza A/Hong Kong/8/68 virus (FLU-HK) for 6 hrs. The expression of pSK1, SK1, and GAPDH was detected by Western blot analysis and intensity of each band was quantified by densitometric analysis. The level of mock-infected pSK1 or SK1 was compared to that of GAPDH and set as 1.0. Relative levels of pSK and SK1 are shown. Values are means ± SEM (n=3). *, p<0.05; **, p<0.01. (B and C) MDCK (B) or HEK293 (C) cells were left untreated or treated with SKI-II (10 µM) or SK1-I (10 µM) and infected with FLU-HK at 1 MOI. Cell lysates were used for Western blot analysis to detect viral proteins M1, M2, NS1, NS2, NP, and α-tubulin at 9 hpi.
(TIF)

Figure S3 **SK inhibitor differentially regulates nuclear export of viral proteins.** (A) MDCK cells were left untreated or treated with DMS (5 µM), SKI-II (10 µM), or SK1-I (10 µM) and infected with influenza virus at 1 MOI. (B) MDCK cells were left untreated or treated with SKI-II (10 µM) upon influenza virus infection at an MOI of 1. Cytosolic and nuclear extracts were isolated from cells at 9 hpi. The expression of viral NP, α-tubulin, and histone H2B was detected by Western blot analysis (top panel). Densitometry was used to compare cytosolic and nuclear levels of viral NP. The relative expression of NP in the cytosol of virus-infected SKI-II-treated cells was compared to the relative expression of NP in the cytosol of virus-infected, untreated cells, which was set at 1.0. Fold changes in the relative ratios are shown (bottom panel). (C and D) MDCK cells were left untreated or treated with DMS (5 µM), SKI-II (10 µM), or LMB (10 ng/mL) and infected with influenza virus at 1 MOI. After staining with antibodies against viral NS2 (C), or M2 (D), viral proteins (green) were visualized by a confocal microscopic analysis. DRAQ5 dye was used to stain nuclei (red). Scale bar = 50 µm.
(TIF)

Figure S4 **A model for the role of SK1 in influenza virus replication.** Influenza virus infection induces activation of SK1. (A) Activated SK1 induces IKK phosphorylation to promote NF-κB translocation into nucleus, leading to the amplification of viral RNAs and proteins required for virus production. (B) Virus-induced activation of SK1 triggers activation of ERK and PI3K/AKT. Activated ERK in turn induces the phosphorylation of p90RSK. p-p90RSK as well as activated AKT elicits RanBP3 phosphorylation, triggering a CRM1/RanBP3-mediated export signal pathway. This will allow the efficient nuclear export of influenza viral RNP complexes into the cytoplasm. Finally, infectious virus particles are released after viral proteins and RNP complex are assembled.
(TIF)
