# Supplementary figures and images for: Sphingosine Kinase 1 Serves as a Pro-Viral Factor by Regulating Viral RNA Synthesis and Nuclear Export of Viral Ribonucleoprotein Complex upon Influenza Virus Infection
Source: PLoS One. 2013 Aug 30;8(8):e75005. doi: 10.1371/journal.pone.0075005 (PMC3796690; doi:10.1371/journal.pone.0075005)

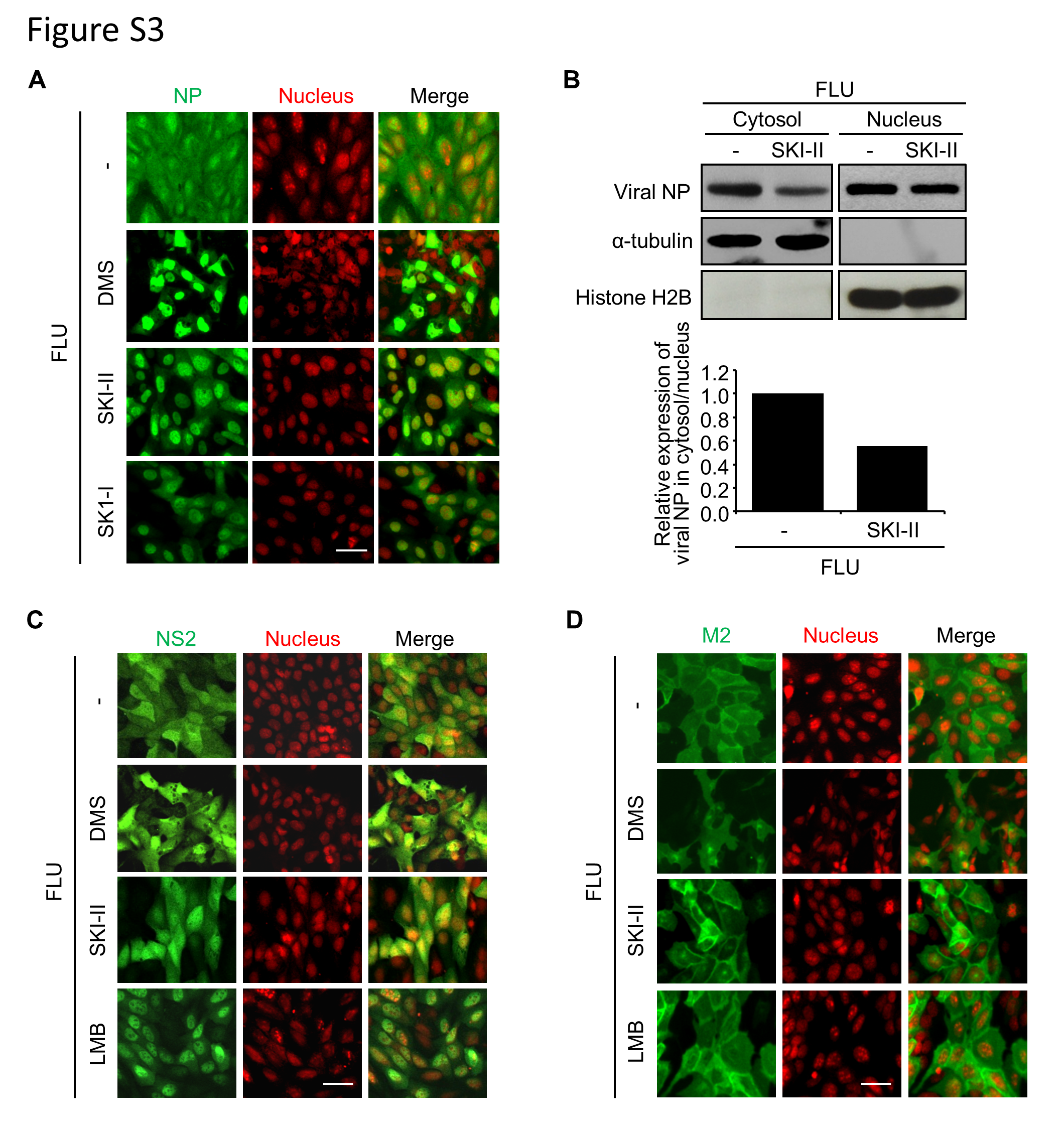

Supplement: Figure S3 — SK inhibitor differentially regulates nuclear export of viral proteins. (A) MDCK cells were left untreated or treated with DMS (5 µM), SKI-II (10 µM), or SK1-I (10 µM) and infected with influenza virus at 1 MOI. (B) MDCK cells were left untreated or treated with SKI-II (10 µM) upon influenza virus infection at an MOI of 1. Cytosolic and nuclear extracts were isolated from cells at 9 hpi. The expression of viral NP, α-tubulin, and histone H2B was detected by Western blot analysis (top panel). Densitometry was used to compare cytosolic and nuclear levels of viral NP. The relative expression of NP in the cytosol of virus-infected SKI-II-treated cells was compared to the relative expression of NP in the cytosol of virus-infected, untreated cells, which was set at 1.0. Fold changes in the relative ratios are shown (bottom panel). (C and D) MDCK cells were left untreated or treated with DMS (5 µM), SKI-II (10 µM), or LMB (10 ng/mL) and infected with influenza virus at 1 MOI. After staining with antibodies against viral NS2 (C), or M2 (D), viral proteins (green) were visualized by a confocal microscopic analysis. DRAQ5 dye was used to stain nuclei (red). Scale bar = 50 µm. (TIF) [file pone.0075005.s003.tif]

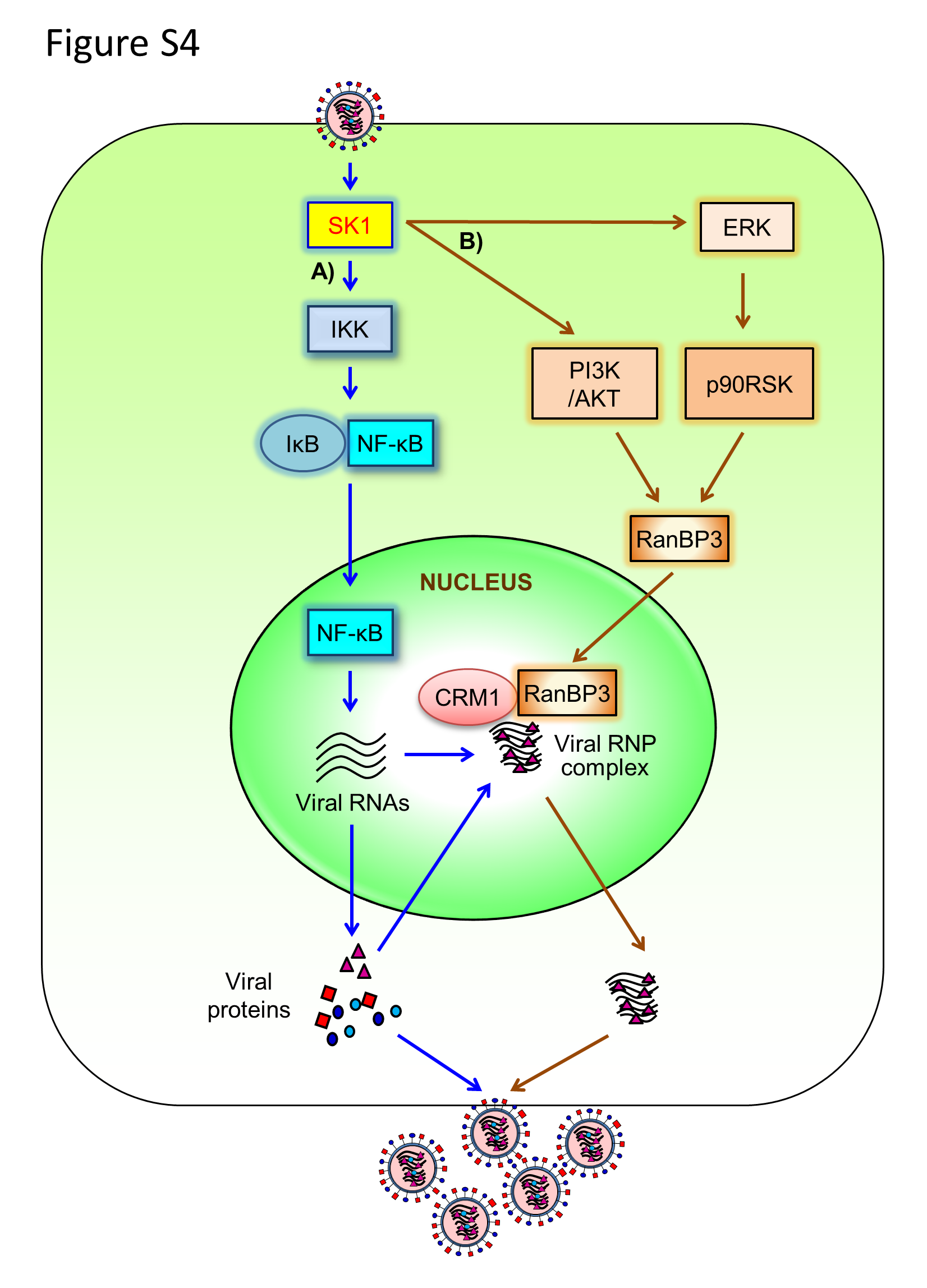

Supplement: Figure S4 — A model for the role of SK1 in influenza virus replication. Influenza virus infection induces activation of SK1. (A) Activated SK1 induces IKK phosphorylation to promote NF-κB translocation into nucleus, leading to the amplification of viral RNAs and proteins required for virus production. (B) Virus-induced activation of SK1 triggers activation of ERK and PI3K/AKT. Activated ERK in turn induces the phosphorylation of p90RSK. p-p90RSK as well as activated AKT elicits RanBP3 phosphorylation, triggering a CRM1/RanBP3-mediated export signal pathway. This will allow the efficient nuclear export of influenza viral RNP complexes into the cytoplasm. Finally, infectious virus particles are released after viral proteins and RNP complex are assembled. (TIF) [file pone.0075005.s004.tif]
